# Clinical Features According to the Type of Intermittent Exotropia: Korean Intermittent Exotropia Multicenter Study

**DOI:** 10.3390/epidemiologia6040068

**Published:** 2025-10-27

**Authors:** Hee Kyung Yang, Hae Ri Yum, Sun A Kim, Hyuna Kim, Jinu Han, Yoonae A. Cho, Hyunkyung Kim, Dong Gyu Choi

**Affiliations:** 1 Department of Ophthalmology, Seoul National University Bundang Hospital, Seoul National University College of Medicine, Seongnam 13620, Republic of Korea; 2 Department of Ophthalmology, Eunpyeong St. Mary’s Hospital, College of Medicine, The Catholic University of Korea, Seoul 03312, Republic of Korea; 3 Sungmo Eye Hospital, Busan 48064, Republic of Korea; 4 Department of Ophthalmology, Soonchunhyang University Seoul Hospital, Seoul 04401, Republic of Korea; 5 Institute of Vision Research, Department of Ophthalmology, Gangnam Severance Hospital, Yonsei University College of Medicine, Seoul 06273, Republic of Korea; 6 Nune Eye Hospital, Seoul 06192, Republic of Korea; 7 Hangil Eye Hospital, Incheon 21388, Republic of Korea; 8 Department of Ophthalmology, Kangnam Sacred Heart Hospital, Hallym University College of Medicine, Seoul 07442, Republic of Korea

**Keywords:** exodeviation, exotropia, multicenter study, near-distance difference, type of exotropia

## Abstract

Background/Objectives: To determine the clinical features of different types of intermittent exotropia according to the distance and near angles of exodeviation. Methods: This study included 5331 patients with intermittent exotropia. The patients were divided into three groups according to the near-distance differences in their exodeviations: (1) Basic-type: difference between distant and near angles of the exodeviation < 10 prism diopters (PD); (2) Convergence insufficiency (CI)-type: near-distance angle ≥ 10 PD; (3) Divergence excess (DE)-type: distance-near angle ≥ 10 PD. The main outcome measures were demographics, clinical characteristics of exotropia, subjective symptoms, medical history, and family history. Results: Overall, 4599 (86.2%) patients had basic-type exotropia, 500 (9.4%) had CI-type, and 232 (4.4%) had DE-type exotropia. Older age and greater magnitude of myopia were associated with CI-type exotropia. A-pattern exotropia, superior oblique (SO) overaction, good fusional control, good stereoacuity, and diplopia were most common in CI-type exotropia. SO underaction and photophobia were most frequently observed in DE-type exotropia compared to the other types. Conclusions: The clinical characteristics varied among the different types of intermittent exotropia. CI-type exotropia was most frequently associated with older age and greater myopia. DE-type exotropia was associated with frequent photophobia.

## 1. Introduction

Intermittent exotropia is the most common type of strabismus in East Asia, including South Korea, as well as in Africa; a higher prevalence has also been found in countries close to the equator [1,2,3,4,5,6,7,8]. The clinical types of intermittent exotropia have been classified based on the difference between the distance and near deviation [9]. This classification is practically important to clinicians for the purposes of surgery, and various studies have reported different clinical features and prognoses after surgery according to the distance/near differences in exodeviation [9,10,11,12,13,14,15,16,17]. For basic-type exotropia, Kushner suggested a unilateral recession–resection procedure [13], which was supported by an 8-year outcomes report from a randomized controlled trial showing better long-term outcomes after unilateral recession–resection for childhood exotropia compared to that for bilateral lateral rectus muscle recession [16]. Bilateral lateral rectus recession with or without augmentation has been preferred for divergence excess (DE)-type exotropia [18,19]; unilateral resection–recession [10], or medial rectus resection have been proposed for convergence insufficiency (CI)-type exotropia [14]. However, most of the studies were conducted in a small number of patients, and few studies reported the types of exotropia in a large study population [20,21,22,23]. This study was conducted as a part of the Korean Intermittent Exotropia Multicenter Study (KIEMS), a nationwide observational study which was performed by the Korean Association for Pediatric Ophthalmology and Strabismus (KAPOS). The KAPOS consists of strabismus specialists throughout the country who provided reliable data from comprehensive ophthalmologic examinations and questionnaires [24,25]. Herein, we determined the prevalence of intermittent exotropia types based on distance and near differences in the angle of exodeviation and compared the clinical features among these types.

## 2. Materials and Methods

### 2.1. Study Design and Participants

We recruited patients with intermittent exotropia at 53 institutions in South Korea from March 2019 to February 2020, and the detailed study protocol has been described in our previous report [24]. A retrospective review was performed using medical records from the KIEMS, including 5331 consecutive patients with sufficient clinical data of intermittent exotropia. Patients with congenital ocular anomalies, incomitant strabismus, ocular myopathies, neurological or paralytic disorders, corneal opacity, cataracts, previous ocular surgery, retinal diseases, and/or blepharoptosis were excluded. The Institutional Review Board of each institution approved this study.

### 2.2. Ophthalmologic Examination

The following data were collected and analyzed: age, sex, refractive errors measured using cycloplegic refraction with 1% cyclopentolate hydrochloride (Cyclogyl, Alcon Lab. Inc., Fort Worth, TX, USA) and 1% tropicamide (Mydriacyl, Alcon Lab. Inc., Fort Worth, TX, USA), best-corrected visual acuity, angles of deviation in the prism and alternate-cover tests (in the primary, secondary, and head-tilted positions under distant [6 m] and near [33 cm] viewing conditions using accommodative targets with the patient’s best optical correction), and associated strabismus. Vertical deviation was defined as hypertropia/hypotropia of ≥5 prism diopters (PD) in the primary position. Lateral incomitance was defined as a decrease in the exodeviation angle of ≥20% in the right or left gaze, as compared with that in the primary position. A-pattern exotropia was defined as a condition in which the exotropia angle at the down gaze was higher by ≥10 PD than that at the up gaze. Likewise, V-pattern exotropia was defined as a condition in which the exotropia angle at the up gaze was higher by ≥15 PD than that at the down gaze. Fusion control was also investigated and classified as follows: good control when ocular fusion was disrupted only after the cover test at distance fixation and was rapidly regained without blinking or fixating ocular movements; fair control, when ocular fusion was regained only after blinking or fixating movements after disruption with cover testing at distance fixation; and poor control, when ocular fusion was spontaneously broken without fusion disruption or was not regained despite blinking or refixation [26]. For sensory status evaluation, the Worth four-dot test (Richmond Products, Albuquerque, NM, USA) under the distance-viewing condition, and either the Titmus stereotest (Stereo Optical Co., Inc., Chicago, IL, USA) or Randot stereotest (Vision Assessment Corporation, Elk Grove Village, IL, USA) under the near-viewing condition (40 cm) were performed. Stereoacuity of ≤60 arcsec in the Titmus stereotest, or ≤63 arcsec in the Randot stereotest, was defined as “good stereopsis.”

### 2.3. Self-Administered Questionnaire

Clinical information regarding subjective symptoms, family history, and medical history was collected from the patients or their guardians using a self-administered questionnaire [24]. Each investigator collected the questionnaires from all patients who met the inclusion criteria [24]. The following information was noted: onset of symptoms; the first person who noticed the associated symptoms; frequency of manifest exotropia noticed per day; guardian’s recognition of exotropia manifestations, such as direction of deviation and fixation dominance [27]; associated symptoms (including abnormal head posture, photophobia, reading difficulty, headache, ocular pain, micropsia, or blurring) [28,29,30]; frequency of diplopia at distance or near-viewing conditions [31]; past medical history of wearing glasses; duration, frequency, and laterality of occlusion therapy [32]; developmental delay, systemic or neurologic diseases, previous surgery; birth history, including type of delivery, gestational age, and birth weight [33]; perinatal medical conditions; and family history of strabismus in parents and/or siblings or history of strabismus surgery in family members [33].

### 2.4. Classification of Exotropia Types According to Distance and near Exodeviation

Patients were divided into three groups according to the near-distance differences in exodeviation: (1) Basic-type: difference between distance and near angles of exodeviation < 10 prism diopters (PD); (2) CI-type: near-distance angle of exodeviation ≥ 10 PD; (3) Divergence excess (DE)-type: distance–near angle of exodeviation ≥ 10 PD [10].

### 2.5. Main Outcome Measures

We compared the following features among the three groups: (1) demographics and ophthalmologic examinations; (2) subjective findings from the self-administered questionnaire; (3) medical history of other diseases and family history of strabismus.

### 2.6. Statistical Analysis

The independent *t* test, likelihood ratio, one-way analysis of variance (ANOVA), and multivariate analysis of variance (MANOVA) were used to compare characteristics among the groups. *p* values < 0.01 were considered statistically significant. Statistical analyses were performed using IBM SPSS Statistics 27.0 (IBM Corporation, Armonk, NY, USA).

## 3. Results

### 3.1. Participants

The demographics and clinical characteristics according to the type of exotropia are shown in Table 1. Among the 5331 patients, basic-type exotropia (difference between distant and near angles < 10 PD), CI-type exotropia (near-distance angle ≥ 10 PD), and DE-type exotropia (distant–near angle ≥ 10 PD) were observed in 4599 (86.2%), 500 (9.4%), and 232 (4.4%) patients, respectively. The sex ratios were not significantly different among the three groups; the proportions of males were 47.7%, 52.8%, and 44.0% in the basic-type, CI-type, and DE-type exotropia groups, respectively (*p* = 0.782, Chi-square test). Median age at the onset of exotropia was 4.0 years (2.0–7.1) in the basic-type, 7.0 years (4.0–9.8) in CI-type, and 3.2 years (2.0–5.7) in DE-type exotropia, and was significantly older in those with CI-type exotropia compared to that in the other groups (*p* < 0.001, one-way ANOVA, Bonferroni-corrected). Median age at diagnosis of exotropia was 6.6 years (4.2–9.1) in the basic-type, 9.0 years (6.8–11.6) in CI-type, and 5.7 (3.2–8.7) in DE-type exotropia, and was significantly older in patients with CI-type exotropia (*p* < 0.001, one-way ANOVA, Bonferroni-corrected).

### 3.2. Ophthalmologic Examination

The mean angles of exodeviation in the primary position at distance and near were 23.7 ± 8.6 PD and 24.9 ± 8.8 PD in the basic-type, 19.6 ± 9.0 PD and 31.3 ± 9.1 PD in CI-type, and 28.1 ± 9.0 PD and 13.2 ± 9.6 PD in DE-type exotropia, respectively. The mean spherical-equivalent refractive errors in the right eye were −0.51 ± 1.87 D in the basic-type, −1.30 ± 2.05 D in CI-type, and −0.26 ± 1.54 D in DE-type exotropia, indicating greater myopia in CI-type exotropia compared to that in the other groups (*p* < 0.001, by one-way ANOVA, Bonferroni-corrected). In patients with CI-type exotropia, A-pattern exotropia (2.5%), superior oblique (SO) overaction (9.3%), good fusional control (38.2%), and good stereoacuity (80.3%) were more frequent than in the other types. Meanwhile, SO underaction was more frequently found in the DE-type (3.2%) than in the CI-type (0.3%) (*p* = 0.001, Chi-square test). (Table 1).

Multivariate analysis revealed that the spherical-equivalent refractive errors in the right eye and left eye were the only significant factors that were different among the groups (*p* = 0.001, *p* = 0.004, MANOVA, respectively).

### 3.3. Self-Administered Questionnaire

The symptoms, according to the type of exotropia, are summarized in Table 2. The frequency of observed squint was less frequent in CI-type (59.0%) than in basic (67.8%) or DE-type exotropia (69.2%) (*p* < 0.001, Chi-square test). Diplopia at near and/or reading difficulty (13.6%), as well as diplopia at distance (11.5%), were more frequently found in CI-type exotropia compared to the basic-type (all *p* < 0.001, Chi-square test). Patients with DE-type exotropia complained of photophobia (58.6%) more than those with CI-type exotropia (49.0%) (*p* < 0.001, chi-square test). There were no differences in abnormal head posture according to the type of exotropia (*p* = 0.383, Chi-square test, Bonferroni-corrected).

The medical and family histories of the patients according to the type of exotropia are summarized in Table 3. The rates of preterm delivery, cesarean section, perinatal diseases, other systemic or neurologic diseases, family history of strabismus, and family history of strabismus surgery were not significantly different among the three groups (all *p* > 0.01, Chi-square test).

## 4. Discussion

In this nationwide observational multicenter study, we determined the prevalence and characteristics of the specific types of intermittent exotropia based on the distance and near differences in exodeviation. This is one of the largest studies, including 5331 patients with intermittent exotropia. It provides reliable objective findings and subjective information obtained from self-administered questionnaires in a large study population. Basic-type exotropia was the most prevalent form (86.2%), followed by the CI-type (9.4%) and DE-type (4.4%) exotropia. CI-type exotropia was associated with older age and greater magnitude of myopia compared to the other types. Good stereoacuity, A-pattern exotropia, SO overaction, near diplopia/reading difficulty, and distant diplopia were most frequently found in CI-type exotropia. SO underaction and photophobia were most frequently observed in patients with DE-type exotropia compared to the other types.

There are few reports regarding the type of exotropia based on distance and near differences including a large number of patients [21,22,23]. In a population-based study by Pan et al. [21], including 5831 preschool Chinese children aged 3–6 years, 166 children had intermittent exotropia, with the basic-type found to be the most common (74.7%), followed by the DE-type (19.9%) and the CI-type (5.4%) exotropia [21]. Despite the small number of patients with exotropia in their study, their findings agreed with ours. In their study, DE-type exotropia was more common, and CI-type exotropia was much less frequent than in our study, which can be explained by the difference in age criteria. In our study, the median age at the onset of DE-type and CI-type exotropia was 3.2 years and 7.0 years, respectively, showing an older age of onset in CI-type exotropia. While most of the previous studies included a small number of patients with exotropia, Wan et al. conducted a large retrospective analysis of 5746 patients with strabismus during a 6-year period in a tertiary eye center in China [22]. Intermittent exotropia was the most common type among the exotropia patients, accounting for 71.3% (2604/3650), and basic-type, CI-type, and DE-type exotropia accounted for 79.8%, 12.1%, and 8.2%, respectively, similar to our results [22]. Interestingly, the differences among the three types over the 6-year study period were statistically significant over time, showing an increase in the proportion of CI-type exotropia from 9.6% (2014–2016) to 13.7% (2017–2019) [22]. In another large retrospective study by Wen et al. [23], including 2250 patients who received strabismus surgery in southern China, basic-type, CI-type, and DE-type exotropia accounted for 79.4%, 11.3%, and 9.3% of the patients, respectively.

In our study, CI-type exotropia was associated with an older age at onset and a greater magnitude of myopia. The greater magnitude of myopia found in CI-type exotropia, to some extent, may be attributed to the older age of onset, as myopia progression continues throughout childhood. The association between myopia and intermittent exotropia has been continuously investigated, showing inconsistent findings, partly because of the various factors affecting myopic progression [23,34,35,36]. In our study, good stereoacuity and diplopia were more frequently found in CI-type exotropia, which is also evidence of good binocular fusion during early childhood prior to the onset of manifest exotropia. The reason for the association of CI-type exotropia with an older age and greater myopia than that of the other types remains unclear. One explanation for this association is the role of accommodative convergence [37,38]. When fusional control deteriorates, increased accommodative demands may be recruited [23,39,40,41], which may play a role in the development and progression of myopia. Meanwhile, accommodative lag is frequently found in myopia, which may induce visual blurring and further deterioration in the fusional vergence system [42]. The decrease in accommodation with age and the higher accommodation lag related to myopia may be partly involved in the uncoupling of accommodation and convergence in CI-type exotropia [43]. Wen et al. [23] reported that CI-type exotropia, or intermittent exotropia with a low accommodative convergence/accommodation ratio, had a greater magnitude of myopia [23]. However, further studies are required to clarify this issue.

Photophobia was more frequently found in DE-type exotropia than CI-type exotropia. Choi et al. [44] reported that transient eye closure (TEC) in response to high-intensity white light was related to self-reported photophobia in patients with intermittent exotropia. In their study, a smaller angle of deviation at near was associated with TEC [44]. This is compatible with our results, as the mean angle of exotropia at near was the smallest in DE-type exotropia (13.2 ± 9.6 PD). TEC under bright light is a form of the photic blink reflex, and the deterioration of fusional amplitude or weakening of binocular sensory status may be triggered by exposure to bright light [28].

Our study has certain limitations. Firstly, the retrospective design of the study may introduce recall bias, inconsistent documentation, rigid classification, limited generalizability, and variability in data-completeness across the institutions. However, these limitations are mitigated by the fact that the KAPOS established a consensus on the standard strabismus examination forms and questionnaires, which were distributed among the members years prior to the study. Secondly, the type of exotropia should be carefully measured, considering the effects of tenacious proximal fusion or accommodative convergence. However, this aspect was not fully evaluated on a regular basis, as data were obtained from multiple centers. Thus, patients presumed to have DE-type exotropia were not thoroughly classified as having pseudo or true DE-type exotropia. Moreover, the type of exotropia may change during repeated examinations or after monocular occlusion [45]. In a previous study, after one day of monocular occlusion in patients with intermittent exotropia, 39.1% of DE-type, 20.0% of CI-type, and 2.7% of basic-type exotropia cases were converted to other types [45]. Even patients with basic-type exotropia may exhibit an increased exodeviation after prolonged occlusion, particularly during near fixation, resulting in the conversion to a CI-type [45]. Regarding the variability in measurements in each visit, and the cross-sectional nature of our study, the results may not reveal the true manifestation or the maximum angle of latent strabismus. A longitudinal follow-up study would thus help to determine the natural course and/or surgical outcomes in this large nationwide cohort. Finally, other important factors, such as genetic background, socioeconomic status, visual habits (including near work and screen time), daily functioning, and psychosocial outcomes, all of which could influence both myopia and exotropia characteristics, were not considered in the study.

## 5. Conclusions

In conclusion, this study is one of the largest to reveal a particular type of intermittent exotropia based on distance and near differences in the angle of exodeviation. While basic-type exotropia was the most prevalent, the clinical characteristics varied among the distinct types of intermittent exotropia. CI-type exotropia was associated with older age of onset, greater myopia, good stereoacuity, and frequent symptoms of diplopia. DE-type exotropia was associated with more photophobia compared to the CI-type. A future longitudinal follow-up study of this large, nationwide cohort could provide valuable insights into the natural course of intermittent exotropia and aid in evaluating treatment outcomes.

## Figures and Tables

**Table 1:** Demographics and clinical characteristics according to the type of exotropia.

	Basic-Type	CI-Type	DE-Type	*p* Value
Number	4599 (86.2%)	500 (9.4%)	232 (4.4%)	
Male	2195 (47.7%)	264 (52.8%)	102 (44.0%)	0.782 ^a^
Onset age (year) ^d^	4.0 (2.0–7.1)	7.0 (4.0–9.8)	3.2 (2.0–5.7) ^d^	<0.001 ^b^, A = C < B ^c^
Age at diagnosis (year) ^d^	6.6 (4.2–9.1)	9.0 (6.8–11.6)	5.7 (3.2–8.7) ^d^	<0.001 ^b^, A = C < B ^c^
Distant exodeviation (PD)	23.7 ± 8.6 (0–85)	19.6 ± 9.0 (0–56)	28.1 ± 9.0 (10–80)	<0.001 ^b^, B < A < C ^c^
Near exodeviation (PD)	24.9 ± 8.8 (0–90)	31.3 ± 9.1 (8–70)	13.2 ± 9.6 (0–50)	<0.001 ^b^, C < A < B ^c^
SEQ Right eye (D)	−0.51 ± 1.87 (−12.88~+7.00)	−1.30 ± 2.05 (−10.25~+4.88)	−0.26 ± 1.54 (−7.50~+3.00)	<0.001 ^b^, B < A = C ^c^
SEQ Left eye (D)	−0.55 ± 1.94 (−14.00~+8.75)	−1.33 ± 2.06 (−9.13~+5.13)	−0.31 ± 1.74 (−7.25~+6.25)	<0.001 ^b^, B < A = C ^c^
IO Overaction	792 (23.1%)	83 (23.0%)	37 (23.0%)	0.999 ^a^
SO Overaction	162 (4.9%)	33 (9.3%)	10 (6.4%)	0.001 ^a^, A < B ^c^
SO Underaction	58 (1.8%)	1 (0.3%)	5 (3.2%)	0.001 ^a^, B < C ^c^
Vertical deviation ≥ 5 PD	236 (5.1%)	31 (6.2%)	11 (4.7%)	0.562 ^a^
Lateral incomitance	89 (2.6%)	19 (4.9%)	1 (0.8%)	0.014 ^a^
A-pattern	24 (0.7%)	10 (2.5%)	1 (0.8%)	0.001 ^a^, A = C < B ^c^
V-pattern	41 (1.2%)	1 (0.3%)	2 (1.6%)	0.202 ^a^
Good Fusion Control *	1114 (26.9%)	174 (38.2%)	41 (20.3%)	<0.001 ^a^, A = C < B ^c^
Good stereoacuity ^†^	2490 (73.5%)	354 (80.3%)	103 (70.5%)	0.006 ^a^, A = C < B ^c^

CI = convergence insufficiency; DE = divergence excess; PD = prism diopters; SEQ = spherical equivalent refractive error; D = diopters; IO = inferior oblique muscle; SO = superior oblique muscle. * When ocular fusion was disrupted only after the cover test at distance fixation and rapidly regained without blinking or fixating ocular movements. ^†^ Stereoacuity of ≤60 arcsec in the Titmus stereotest or ≤63 in the Randot stereotest. ^a^ Pearson’s Chi-square test; ^b^ One-way analysis of variance, ^c^ Post hoc test by Bonferroni (A = Basic-type, B = CI-type, C = DE-type), ^d^ Median (25th percentile–75th percentile).

**Table 2:** Subjective findings from self-administered questionnaires according to the type of exotropia.

	Basic-Type	CI-Type	DE-Type	*p* Value
Squint observed	2925 (67.8%)	275 (59.0%)	155 (69.2%)	0.001 ^a^, B < A = C ^b^
Frequency ≥ 1/day	2721 (68.2%)	252 (59.6%)	153 (73.6%)	<0.001 ^a^, B < A = C ^b^
Abnormal head posture	121 (7.8%)	13 (7.6%)	4 (4.9%)	0.383 ^a^
Near diplopia or Reading difficulty	314 (7.3%)	64 (13.6%)	16 (7.6%)	<0.001 ^a^, A < B ^b^
Distant diplopia	262 (6.1%)	54 (11.5%)	16 (7.6%)	<0.001 ^a^, A < B ^b^
Photophobia	2392 (52.0%)	245 (49.0%)	136 (58.6%)	<0.001 ^a^, B < C ^b^

CI = convergence insufficiency; DE = divergence excess. ^a^ Pearson’s Chi-square test; ^b^ Post hoc test by Bonferroni (A = Basic-type, B = CI-type, C = DE-type).

**Table 3:** Medical and family history according to the type of exotropia.

	Basic-Type	CI-Type	DE-Type	*p* Value
Preterm delivery	409 (9.0%)	37 (7.4%)	23 (10.1%)	0.360 ^a^
Cesarean section	1646 (41.1%)	161 (38.6%)	99 (47.4%)	0.085 ^a^
Perinatal disease	193 (4.7%)	17 (4.0%)	7 (3.4%)	0.530 ^a^
Other disease ^b^	480 (10.7%)	41 (8.4%)	20 (8.8%)	0.194 ^a^
Family history of strabismus	685 (14.9%)	70 (14.0%)	36 (15.5%)	0.829 ^a^
Family history of strabismus surgery	245 (5.3%)	32 (6.4%)	17 (7.3%)	0.283 ^a^

CI = convergence insufficiency; DE = divergence excess. ^a^ Pearson’s Chi-square test; ^b^ Developmental delay, or systemic or neurologic diseases.

## Data Availability

The Institutional Review Board of Seoul National University Bundang Hospital/Ethics Committee has placed ethical restrictions to protect patient identities. However, the data is available to anyone who is interested without restriction. The minimal data set will be available upon request. For data requests, please contact the SNUBH IRB office at 82-31-787-8804, 98614@snubh.org.

## Abbreviations

The following abbreviations are used in this manuscript:

CI	convergence insufficiency
DE	divergence excess
KIEMS	Korean Intermittent Exotropia Multicenter Study
KAPOS	Korean Association for Pediatric Ophthalmology and Strabismus
PD	prism diopters
ANOVA	one-way analysis of variance
MANOVA	multivariate analysis of variance
SO	superior oblique
TEC	transient eye closure
